# Differential expression of MUC genes in endometrial and cervical tissues and tumors

**DOI:** 10.1186/1471-2407-5-124

**Published:** 2005-09-27

**Authors:** Vidya Hebbar, Gautam Damera, Goverdhan P Sachdev

**Affiliations:** 1 Department of Pharmaceutical Sciences, College of Pharmacy, University of Oklahoma Health Sciences Center, P.O. Box 26901, Oklahoma City, OK- 73190, USA; 2 Department of Pharmaceutics, Ernest Mario School of Pharmacy, Rutgers, The State University of New Jersey, 160 Frelinghuysen Road, Piscataway, NJ-08854, USA

## Abstract

**Background:**

Mucin glycoprotein's are major components of mucus and are considered an important class of tumor associated antigens. The objective of this study was to investigate the expression of human *MUC *genes (*MUC1*, *MUC2*, *MUC5B*, *MUC5AC and MUC8*) in human endometrium and cervix, and to compare and quantitate the expression of *MUC *genes in normal and cancerous tissues.

**Methods:**

Slot blot techniques were used to study the *MUC *gene expression and quantitation.

**Results:**

Of the five-mucin genes studied, *MUC1*, *MUC5B *and *MUC8 *showed high expression levels in the normal and cancerous endometrial and cervical tissues, *MUC2 *and *MUC5AC *showed considerably lower expression. Statistically, higher levels of *MUC1*, *MUC5B *and *MUC8 *were observed in endometrial adenocarcinomas compared to normal tissues. In contrast, only *MUC1 *levels increased with no significant changes in expression of *MUC5B *and *MUC8 *in cervical tumors over normal cervical tissues.

**Conclusion:**

Endometrial tumors showed increased expression of *MUC1*, *MUC5B *and *MUC8 *over normal tissues. Only *MUC1 *appears to be increase, in cervical tumors. All the studied tissues showed high and consistent expression of *MUC8 *mRNA. Low to neglible levels of *MUC2 *and *MUC5AC *were observed in all studied endometrial and cervical tissues.

## Background

Mucins are high molecular weight glycoprotein components of mucus (> 250 kDa), which protect and lubricate the epithelial surfaces of the respiratory, gastrointestinal and reproductive tracts in the body [1]. Mucins are heavily glycosylated (40–80%) and the oligosaccharides are attached through O-glycosidic linkages to the hydroxyl group of serine and threonine in the protein backbone [2,3]. The striking feature of nearly all mucin genes isolated thus far is the presence of repeat sequences that are either tandem in nature as in the case of *MUC1 *[4] or slightly imperfect repeats as in the case of *MUC8 *[5]. In general, the repeats are found in the central portion of the protein backbone, which are flanked by unique regions. Mucins have been classified as either membrane-bound or secretory depending on the presence of a putative trans-membrane region.

Altered mucin secretions and/or *MUC *gene expression patterns have been implicated in several cancerous conditions, such as gastric carcinomas [6-10], colorectal carcinomas [11-13], breast cancers [14-16] esophageal carcinomas [17], pancreatic tumors [18-20] and lung adenocarcinomas [21-23]. Accordingly, studies have also been conducted to determine the expression of *MUC *genes in human reproductive tissues and to investigate their possible altered quantitative and/or qualitative expression in cancerous conditions. Serial analysis of gene expression of ovarian cell lines and tissues indicated that among other genes, *MUC1 *is up-regulated in cancerous conditions [24]. Another study also showed that the expression of *MUC1 *correlated with poor prognosis in ovarian carcinoma [25]. Normal endocervical epithelium was found to express *MUC *genes *1*, *4*, *5AC*, *5B*, and *6*, with relatively weak expression of *MUC2*. Normal endocervical and vaginal epithelium expresses *MUC *genes *1 *and *4 *and endometrial epithelium expresses *MUC1 *and *MUC6 *[26,27]. In an earlier report, we demonstrated the antigenic similarities between respiratory and reproductive tract mucins using a variety of mucin antibodies [28]. In another study, we reported the antigenic cross reactivity of human tracheal mucin with male and female reproductive tissues expressing *MUC8 *mRNA[29]. While these reports, in general, indicate the manifestation of a variety of *MUC *genes in reproductive tissues, information regarding mucin gene expression in corresponding tumor tissues is still lacking.

According to The American Cancer Society's Cancer Facts and Figures, it is estimated that there will be 12,200 new cases of invasive cervical cancer (uterine cervix) and 40,100 new cases of endometrial cancer (uterine corpus) that will be diagnosed this year. Hence, a better understanding of *MUC *gene expression patterns in female reproductive malignancies would help enhance prognosis and therapy. To contribute towards this purpose, we investigated the expression of five mucin genes (*MUC1*, *MUC2*, *MUC5AC*, *MUC5B *and *MUC8*) in normal reproductive and cancerous tissues.

## Methods

### Human tissues

Normal and malignant endometrium and cervical tissues were obtained from Co-operative Human Tissue Network (CHTN, Birmingham, AL) and National Disease Research Interchange (NDRI, Philadelphia, PA). Malignant tissues with over 95 percent tumor content were selected for the study. The nomenclature adopted for the tissues was as follows: endometrial adenocarcinomas (EA), normal endometrium (EN), cervical carcinomas (CA), normal cervix (CX). These tissues were acquired on dry ice and kept frozen at -80°C until use. The classification of tumors was performed according to the International Federation of Gynecology and Obstetrics (FIGO) as indicated in Table 2.

The normal tissues EN (mean age: 41, median age: 43) and CX (mean age: 43, median age: 44) used in the study were obtained by hysterectomy for benign gynecological disorders. The menstrual cycle of all the patients with normal tissues were in early to late proliferative phases, except for EN3, EN4, EN10, CX3, CX4 and CX13 were in late secretory phase. The provided pathology reports of both normal and tumor tissues indicated no inflammation or infections.

### Total RNA isolation

Total RNA was extracted from frozen tissues using the TRIzol reagent (Life Technologies) according to manufacturer's protocol. Briefly, endometrial and cervical tissues were ground using a mortar and pestle under liquid nitrogen. The ground tissue was transferred to tubes containing appropriate amounts of TRIzol reagent and homogenized using a Brinkmann homogenizer. The mixture was allowed to set for 5–10 min followed by the addition of appropriate amount of chloroform. This mixture was shaken vigorously and centrifuged at 8,000 × *g *for 30 min. The aqueous layer was collected and the RNA precipitated using isopropanol. The RNA pellet was washed using 70% alcohol and dissolved in RNase free water. To determine the quality of extracted RNA, the dissolved samples were elecrophoresed on 1% formaldehyde-agarose gels to check the integrity of 18s and 28s bands. Samples with OD 260/280 greater than 1.50 were used in the study.

### Slot blot analyses

Total RNA (10 μg) extracted from tissues was blotted directly onto nylon membranes using a Hoefer PR 648 slot blot manifold (Amersham Biosciences, San Francisco, CA). The membranes were pre-hybridized in 5× SSPE, 5× Denhardt's solution, 0.5% SDS, 50% formamide and 40 μg/ml salmon sperm DNA for 12 h at 42°C. The cDNA probes (*MUC1*, *MUC2*, *MUC5B*, *MUC5AC*, *MUC8 *and β-*actin*) were labeled by the random priming technique using DECAprime II kit (Ambion, Austin, TX). Hybridization of ^32^P-labeled cDNA probes was carried out at 42°C for 14–16 h. After hybridization, membranes were washed twice in 2× SSC and 0.1% SDS for 10 min. at room temperature, followed by two additional washes for 20 min at 65°C in 1× SSC and 0.1% SDS solution. The membranes were subsequently exposed to Kodak X-Omat AR films at -70°C. The films were scanned using a Personal SI Densitometer (Molecular Dynamics, Sunnyvale, CA). Following exposure, the membranes were stripped with 0.5% SDS and re-probed with β-actin. Densitrometric units were calculated for each sample after normalization of the readings with corresponding densitrometric readings obtained using β-actin cDNA probe. The hybridization experiments on total RNA from the tissues was performed in triplicate for statistical analyses.

### Source of mucin cDNA probes

The cDNA probes used in the study were designed to exclude the VNTR regions of the studied *MUC *genes. This step was deemed essential to avoid the differences in the numbers of tandem repeats commonly associated with mucins among different individuals. The cDNA probes for *MUC1 *(~500 bp) was kindly provided by Dr. Sandra Gendler [30], *MUC2 *(~450 bp) was a kind gift from Dr. James Gum [31], *MUC5AC *(~800 bp) and *MUC5B *(~340 bp) were generated in the laboratory using specific primers to the published sequences. Earlier, *MUC8 *cDNA probe (1.4 kb) sequence has been reported from our laboratory [5]. This sequence was used in this study to develop a non-repeat 195 bp *MUC8 *cDNA probe. A 1.1 kb cDNA probe specific for β-actin was used for the analyses of housekeeping gene.

### Reverse Transcription and Polymerase Chain Reaction (RT-PCR)

Five micrograms of total RNA was reverse transcribed to cDNA by 200 units Superscript II reverse transcriptase (Life Technologies). The reaction mixture was then treated with 2U RNaseH at 37°C for 20 min and stored at -20°C. Twenty percent of the first strand cDNA was used for the PCR amplification. Primers and annealing temperatures used for RT-PCR are summarized in Table 1.

### Statistical analyses

The data obtained from slot blot analyses of *MUC *genes in both normal and tumor tissues were subjected to parametric statistical analysis using SAS software (SAS Institute, Cary, NC). Two tailed unequal variance student *t *test was used to establish statistical significance. A p value of less than 0.05 was considered significant.

## Results

### Quantitation of mucin gene expression in endometrial tissues

**Figure 1:**
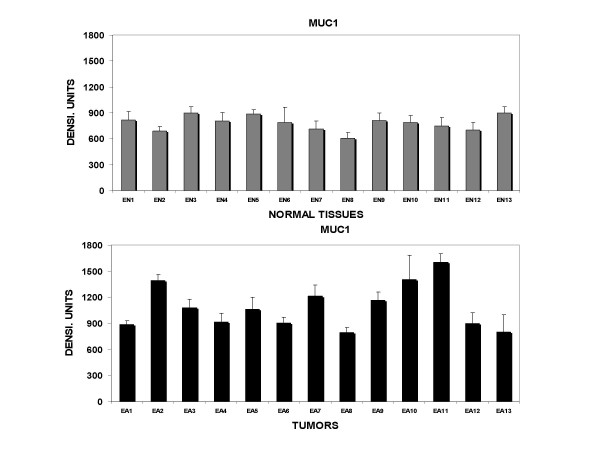
Slot blot analyses of total RNA from endometrial normal (EN) and tumors (EA) tissues using mucin *MUC1 *cDNA probe. Each graph is representative of three experimental replicates. Data normalized using β-*actin *as described in Methods.

**Figure 2:**
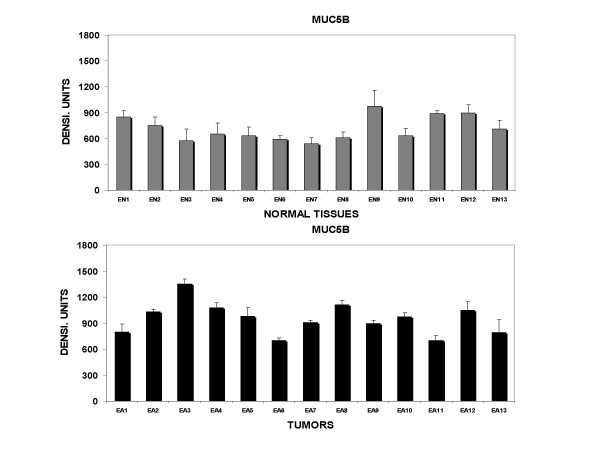
Slot blot analyses of total RNA from endometrial normal (EN) and tumors (EA) tissues using mucin *MUC5B *cDNA probe. The data was normalized using β-*actin *as described in Methods section.

**Figure 3:**
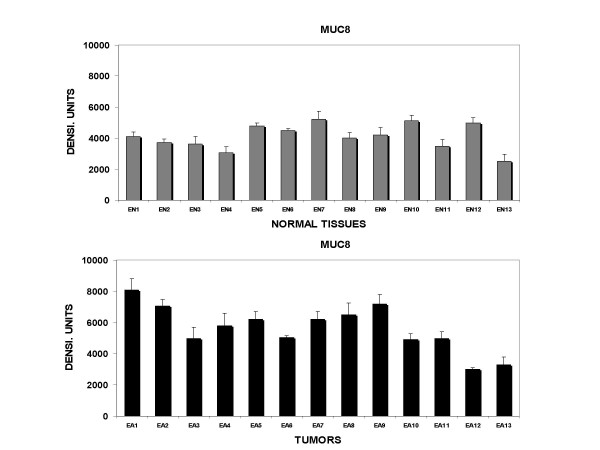
Slot blot analyses of total RNA from endometrial normal (EN) and tumors (EA) tissues using mucin *MUC8 *cDNA probe. The data was normalized using β-*actin*. Note: Larger scale used, indicative of higher expression of *MUC8*.

As shown in Fig. 1*MUC1 *expression was significantly lower in the normal endometrium as compared to the endometrial adenocarcinomas (p = 0.001). Expression of *MUC5B *followed a similar pattern with higher expression observed in 8 out of 13 endometrial tumor tissues studied over normal tissues (Fig. 2). The differences in *MUC5B *levels between the cancerous and non-cancerous endometrial samples was significant (p = 0.006). On the other hand, expression of *MUC8 *was high in all endometrial tissues, with the expression in endometrial adenocarcinomas being significantly higher than the normal endometrium (p = 0.003) (Fig. 3). The box plot analyses showing the relationship between *MUC *gene expression in endometrial tumor and normal tissues are depicted in Fig. 7. No appreciable expression of *MUC2 *and *MUC5AC *in normal and tumor tissues was detected by slot blot analyses.

### Quantitation of mucin gene expression in cervical tissues

**Figure 4:**
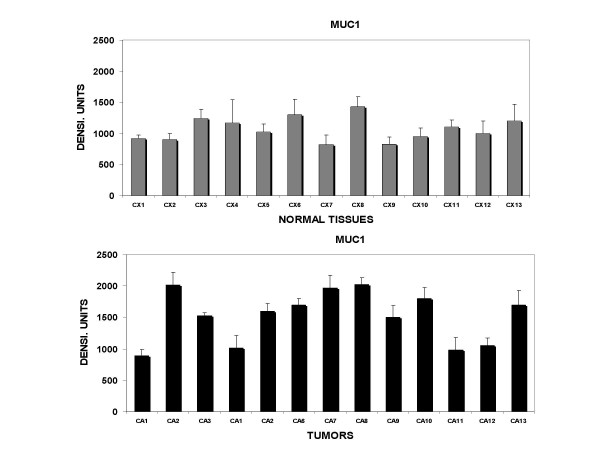
Analyses of total RNA from cervical normal (CN) and tumors (CA) tissues using *MUC1 *cDNA probe. Data was normalized using β-*actin *cDNA probe.

**Figure 5:**
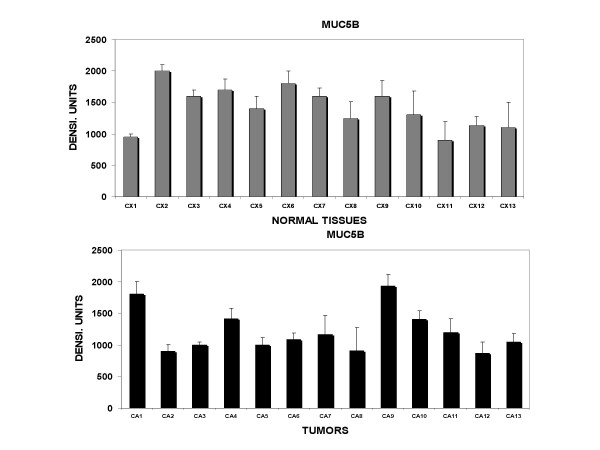
Analyses of total RNA from cervical normal (CN) and tumors (CA) tissues using *MUC5B *cDNA probe. Data was normalized using β-*actin *as described in Methods section.

**Figure 7:**
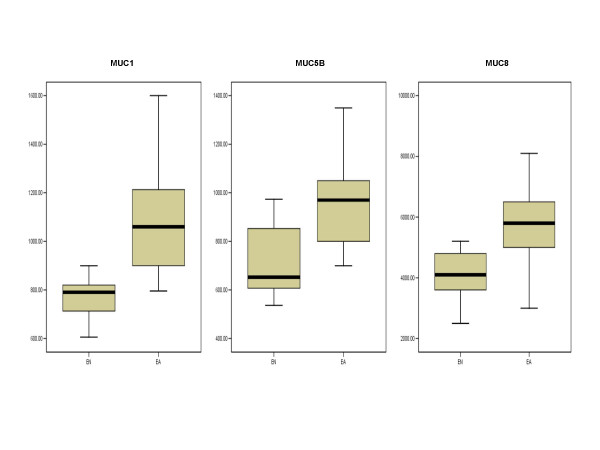
Box plots showing the relationship between *MUC *gene expression in endometrial tumors tissues (EA) and normal tissues (EN). (a) *MUC1 *(b) *MUC5B *(c) *MUC8*.

**Figure 8:**
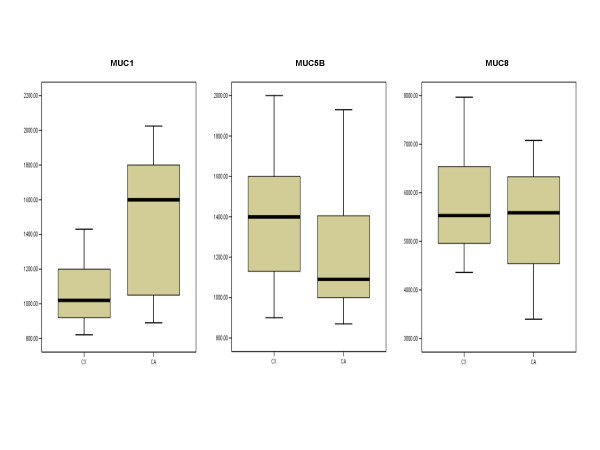
Box plots showing the relationship between *MUC *gene expression in cervical tumors tissues (CA) and normal tissues (CN). (a) *MUC1 *(b) *MUC5B *(c) *MUC8*.

Levels of expression of *MUC1 *in cervical carcinomas were significantly different from normal cervical tissue. (p = 0.002) (Fig. 4). While the expression of *MUC5B*, was higher than *MUC1 *in normal cervical tissues, no statistical significance was observed in *MUC5B *levels between normal and cancerous cervical tissues (p = 0.14) (Fig. 5). The expression *MUC8 *mRNA, were high in all cervical tissues with no statistical difference observed in expression between cancerous and non-cancerous tissues (p = 0.5). Also, box plots depicting MUC gene expression in cervical tumor and normal tissues are illustrated in Fig. 8.

## Discussion

This study is primarily focused on the quantitating the expression of five mucin genes, namely, *MUC1*, *MUC2*, *MUC5AC*, *MUC5B *and *MUC8 *in normal human endometrial and cervical tissues and respective tumors. Studies in tumors have suggested that mucins are aberrantly expressed in cancerous conditions. In the present investigation we observed that of the five *MUC *genes studied, *MUC1*, *MUC5B *and *MUC8 *were expressed at higher levels than *MUC2 *and *MUC5AC *in endometrial and cervical tissues. Similar studies by other investigators on endocervical tissues have revealed *MUC*s *1*, *4*, *5AC*, *5B *and *6 *were expressed at high levels with very low expression of *MUC*s *2*, *3 *and *7 *[26]. However, a follow-up study to this work quantifying the expression of these *MUC *genes in normal endocervical epithelium revealed that *MUC4 *and *MUC5B *were predominantly expressed as compared to *MUC6 *and *MUC5AC *[32]. While acknowledging the variations in the analyzed tissue types, it appears that expression patterns of *MUC2 *and *MUC5AC *genes in this study are broadly confirmatory with earlier reports.

Human endometrial epithelium undergoes progesterone-modulated differentiation during menstrual cycle [33,34]. The qualitative and quantitative changes in the secretion of the endometrium are associated with the proliferation of glandular epithelium with increased golgi and secretory apparatus [35]. Accordingly, mucin secretions and *MUC *gene expression of the female reproductive tissues are dependant on the stage of the menstrual cycle[36]. Earlier studies have shown that *MUC1 *expression in endometrial tissues is at the highest in early to mid secretory phases [37]. To minimize the ambiguity of elevated *MUC *gene expression due to menstrual cycle, the tissues studied in the present investigation are from patients in either proliferative or late secretory phases. Mucin gene regulation and expression has been associated with the effects of immune cell derived inflammatory mediators [38] and bacterial endotoxins [39] on the secretory epithelium in various chronic diseases states of human body. However, in this study the pathology reports of the obtained tissues indicate no inflammation or infection thus minimizing the effect of these mediators on overall results of this study.

MUC1, a trans-membrane mucin, has been studied extensively in the female reproductive tract and is expressed in the endometrium. It plays an important role during implantation and maintenance of the embryo [40,41]. Our studies revealed high *MUC1 *levels in endometrial adenocarcinomas and cervical carcinomas when compared to the normal endometrial and cervical tissues. These results are in accord with studies where *MUC1 *up-regulation was reported in variety of carcinomas including pancreas[42], breast[43], stomach[44], colon rectum [45] and lung [46].

In addition, to altered gene regulation patterns in cancerous conditions, it is reported that alterations may exist in the carbohydrate structures attached to the protein backbone or in the protein backbone itself. In MUC1, alteration in glycosylation patterns leads to tumor-specific peptide epitopes that are exposed in cancerous cells [47]. In breast cancer tissue, an alternatively spliced form of *MUC1*, which is completely devoid of repeats was observed [14]. This variant of *MUC1 *was not expressed in adjacent normal breast tissue. Such studies and others indicate the importance of mucins as tumor markers for diagnostic as well as for therapeutic purposes. For example, in ovarian cancers the CA125 antigen is routinely used to monitor the progress of patients. Recently, investigators found that the CA125 antigen does indeed belong to the mucin family of genes and its core was identified and designated as MUC16 [48].

Our laboratory has previously reported a novel mucin gene *MUC8 *from human normal tracheal tissue [5] and here we have studied the expression of *MUC8 *in human normal as well as cancerous endometrial and cervical tissues. Our earlier studies demonstrated that *MUC8 *is expressed in the male and female reproductive tract [29]. The present work has led us to believe that *MUC8 *is a major mucin expressed in the female reproductive tract. Levels of *MUC8 *are significantly higher in the endometrial adenocarcinomas as compared to the normal endometrium. Although the expression of *MUC8 *was very high in the cervical tissues, no difference in expression was observed among the cancerous and non-cancerous tissues. In the context of *MUC *gene expression, this is the first study reporting differential expression of *MUC8 *and *MUC5B *in cervical and endometrial tissues.

One significant conclusion that can be drawn from this study is that mucin genes, *MUC1*, *MUC5B *and *MUC8 *are all up-regulated in endometrial adenocarcinomas. Furthermore, *MUC2 *and *MUC5AC *were found to be expressed at extremely low levels in the endometrial and cervical tissues studied. In conclusion, this study provides significant information on mucin genes in the female reproductive tract and attempts to understand if there are disease-related changes that mucin genes may undergo in endometrial and cervical carcinomas.

## Competing interests

The author(s) declare that they have no competing interests.

## Authors' contributions

VH and GD carried out gene expression studies and performed statistical analysis. GPS conceived and coordinated the study.

## Pre-publication history

The pre-publication history for this paper can be accessed here:



## Figures and Tables

**Figure 6:**
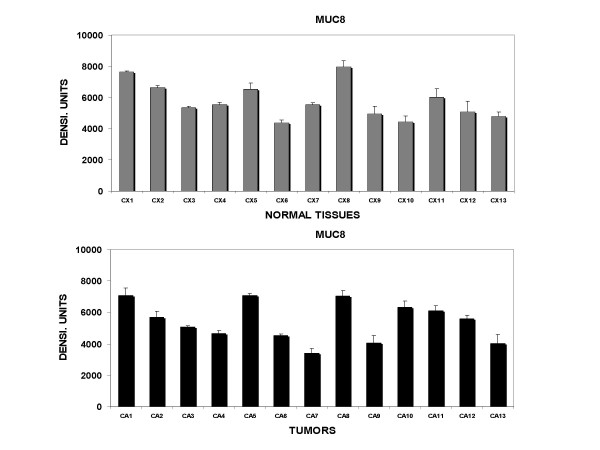
Analyses of total RNA from cervical normal (CN) and tumors (CA) using *MUC8 *cDNA probe. Data was normalized using β-*actin *as described in Methods section. Note: Larger scale used, indicative of higher expression of *MUC8*.

**Table 1:** Primers, annealing temperatures and accession numbers for *MUC *genes

Gene	Acc. #	Primers	A.temp
*MUC5AC*	AF015521	S-GTGGAACCACGATGACAGC	60°C
		AS-TCAGCACATAGCTGCAGTCG	
*MUC5B*	Y09788	S-TGCAATCAGCACTGTGACATTGAC	60°C
		AS-TTCTCCAGGGTCCAGGTCTCATTC	
MUC8	U14383	S-GTTCAGGTCTCCTGCCGG	57°C
		AS-CGAGGCGCCATCATGGAC	

Acc. # are obtained from GenBank provided by National Center for Biotechnology Information, Bethesda, MD.

**Table 2:** Tissue classification of endometrial (EA) and cervical (CA) carcinomas.

Tissue	Age	Grade	Differentiation
EA1	44	II	wd
EA2	47	II	md
EA3	50	I	wd
EA4	48	III	pd
EA5	38	III	pd
EA6	53	I-II	md
EA7	45	III	wd
EA8	54	I	md
EA9	58	II	md
EA10	44	III	md
EA11	36	I	wd
EA12	50	II	md
EA13	31	I	md
CA1	30	IB	md
CA2	35	IB	pd
CA3	48	IB	pd
CA4	39	IB	md
CA5	63	III	pd
CA6	46	II	wd
CA7	57	II	md
CA8	40	IIB	pd
CA9	33	I	md
CA10	42	I	pd
CA11	52	I-II	wd
CA12	31	II	pd
CA13	47	II	md

pd-poorly differentiated, md-moderately differentiated and wd-well differentiated
